# Rare copy number variants contribute pathogenic alleles in patients with intestinal malrotation

**DOI:** 10.1002/mgg3.549

**Published:** 2019-01-10

**Authors:** Karin Salehi Karlslätt, Maria Pettersson, Nina Jäntti, Przemyslaw Szafranski, Tomas Wester, Britt Husberg, Ulla Ullberg, Pawel Stankiewicz, Ann Nordgren, Johanna Lundin, Anna Lindstrand, Agneta Nordenskjöld

**Affiliations:** ^1^ Department of Women’s and Children’s Health and Center for Molecular Medicine Karolinska Institutet Stockholm Sweden; ^2^ Department of Pediatrics Karolinska University Hospital Stockholm Sweden; ^3^ Department of Molecular Medicine and Surgery and Center for Molecular Medicine Karolinska Institutet Stockholm Sweden; ^4^ Department of Clinical Genetics Karolinska University Hospital Stockholm Sweden; ^5^ Department of Molecular and Human Genetics Baylor College of Medicine Houston Texas; ^6^ Department of Pediatric Surgery Karolinska University Hospital Stockholm Sweden; ^7^ Department of Women’s and Children’s Health Karolinska Institutet Stockholm Sweden; ^8^ Department of General Surgery Ersta Hospital Stockholm Sweden; ^9^ Department of Pediatric Radiology Karolinska University Hospital Stockholm Sweden

**Keywords:** array‐CGH, copy number variants, genetic, intestinal malrotation, intestinal rotation abnormalities, midgut volvulus

## Abstract

**Background:**

Intestinal malrotation is a potentially life‐threatening congenital anomaly due to the risk of developing midgut volvulus. The reported incidence is 0.2%–1% and both apparently hereditary and sporadic cases have been reported. Intestinal malrotation is associated with a few syndromes with known genotype but the genetic contribution in isolated intestinal malrotation has not yet been reported. Rare copy number variants (CNVs) have been implicated in many congenital anomalies, and hence we sought to investigate the potential contribution of rare CNVs in intestinal malrotation.

**Methods:**

Analysis of array comparative genomic hybridization (aCGH) data from 47 patients with symptomatic intestinal malrotation was performed.

**Results:**

We identified six rare CNVs in five patients. Five CNVs involved syndrome loci: 7q11.23 microduplication, 16p13.11 microduplication, 18q terminal deletion, *HDAC8* (Cornelia de Lange syndrome type 5 and *FOXF1*) as well as one intragenic deletion in *GALNT14*, not previously implicated in human disease.

**Conclusion:**

In the present study, we identified rare CNVs contributing pathogenic or potentially pathogenic alleles in five patients with syndromic intestinal malrotation, suggesting that CNV screening is indicated in intestinal malrotation with associated malformations or neurological involvements. In addition, we identified intestinal malrotation in two known syndromes (Cornelia de Lange type 5 and 18q terminal deletion syndrome) that has not previously been associated with gastrointestinal malformations.

## 1 INTRODUCTION

Intestinal malrotation is a potentially life‐threatening congenital anomaly. It results from the incomplete rotation and fixation of the gut during embryological and fetal development. The incidence is reported to be 0.2% in the general population based on radiological studies, though autopsy studies estimate a true incidence of 1% (McVay, Kokoska, Jackson, & Smith, 2007).

During normal fetal development, the midgut develops as a loop with the mesenteric artery as an axis. During the fourth fetal week, the midgut will grow and differentiate to the distal duodenum, jejunum, ileum, cecum, and the first part of the colon. The intestines will herniate into the base of the umbilicus during sixth week of gestation due to the small fetal abdomen. During 10th week of gestation, the intestines return back into the abdominal cavity and the abdomen will expand. In this process, the intestines rotate 270° counterclockwise and when folding back into the abdomen, it fixates against the posterior abdominal wall. A disturbance of this process causes intestinal malrotation. (Martin & Shaw‐Smith, 2010; McVay et al., 2007) Intestinal malrotation can also be a secondary phenomenon due to other malformations, like congenital diaphragmatic hernia, gastroschisis or omphalocele, not permitting a normal intestinal rotation. Dysmotility of the small intestine has been associated with intestinal malrotation (Coombs, Buick, Gornall, Corkery, & Booth, 1991; Devane et al., 1992; Erez et al., 2001; Husberg et al., 2016).

Intestinal malrotation lacking a proper fixation of the intestines and a thin mesenteric base is per see a condition without symptoms, but predisposes to midgut volvulus (Ezer et al., 2016; Millar, Rode, & Cywes, 2003) and can cause duodenal obstruction and strangulation of the circulation in the superior mesenteric vessels. Other milder chronic symptoms may occur from duodenal obstruction caused by Ladd's bands overriding the duodenum from cecum to the abdominal posterior wall that causes failure to thrive due to difficulties to accept larger meals or vomiting (Ezer et al., 2016). Midgut volvulus requires emergency surgery to reduce the volvulus, as well as Ladd's procedure to treat the occurrence of malrotation. The small intestines are thus placed to the right and the large intestines to the left part of the abdomen (Swenson & Ladd, 1945). The mesentery is widened to avoid relapse, and the Ladd's bands are divided. This procedure is also done as elective surgery in patients with more chronic clinical symptoms from intestinal malrotation.

The pathogenesis of intestinal malrotation is not known, and most cases are isolated and sporadic however familial cases have been reported (Beaudoin, Mathiot‐Gavarin, Gouizi, & Bargy, 2005; Smith, 1972), mainly in siblings. Generally, malrotation is regarded as a complex disorder with a genetic as well as an environmental background (Martin & Shaw‐Smith, 2010). In the past decade, genomic deletions and duplications collectively called copy number variants (CNVs) have been implicated in many different malformation syndromes (Hofmeister et al., 2018; Lindstrand et al., 2014; Winberg et al., 2014). Heterotaxy syndrome (MIM#306955) is frequently associated with intestinal malrotation and deletions in heterotaxy syndrome‐associated genes *CFC1* (MIM#605194) and *FOXA2* (MIM#600288) have been described as disease‐causing (Cao et al., 2015; Tsai et al., 2015). Further, deletions encompassing *FOXF1* (MIM#601089) is a recognized cause of alveolar capillary dysplasia with misalignment of pulmonary veins (MIM#265380) (Stankiewicz et al., 2009), where intestinal malrotation is common (Sen, Thakur, Stockton, Langston, & Bejjani, 2004). Finally, genes involved in cilia formation are an established cause of left‐right asymmetry (Dasgupta and Amack, 2016) and Jeune syndrome (MIM#208500), a highly heterogeneous ciliopathy, is commonly associated with intestinal malrotation (Hall et al., 2009).

Given this, we screened a cohort of intestinal malrotation patients for rare CNVs. To allow for detection of both large (>100 kb) and small (<10 kb) CNVs, we used a custom‐designed array comparative genomic hybridization (aCGH), with exon resolution in genes involved in known malformation syndromes and the cilia proteome. The findings show a high frequency (7.1%) of likely disease‐causing CNVs in intestinal malrotation patients. Our study indicates that rare CNVs may be involved in the formation of intestinal malrotation and that genetic analysis with CNV screening is indicated in patients with intestinal malrotation, especially if extra‐gastrointestinal malformations and/or neurological involvement are present.

## 2 MATERIALS AND METHODS

### 2.1 Ethical compliance

The study design is in accordance with the Declaration of Helsinki/GCP and approved by the Regional Ethical Review Board in Stockholm, Sweden. Written‐informed consent was collected from all participants or parents/legal guardians of participants.

### 2.2 Study subjects and clinical assessment

**Figure 1:**
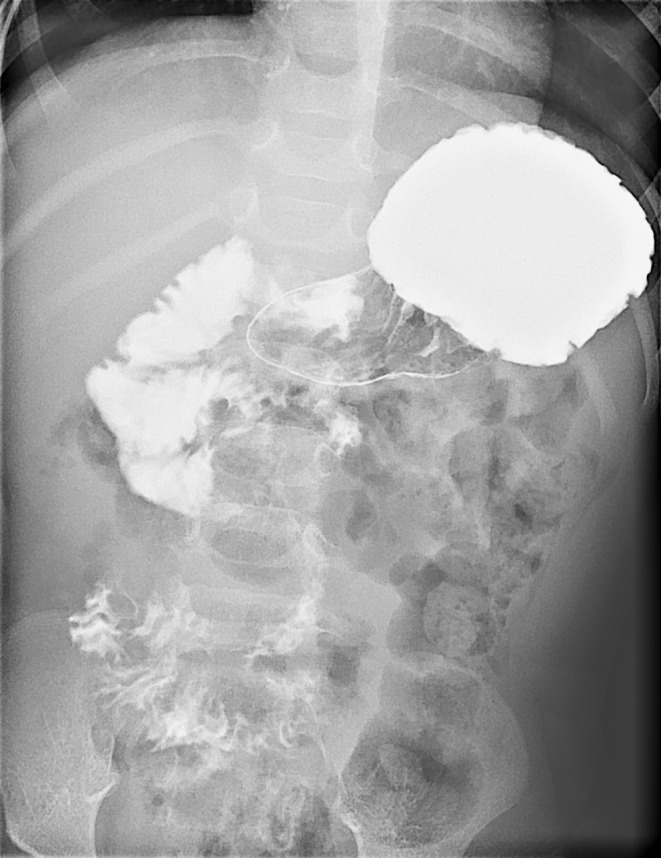
Upper gastrointestinal study of patient 3, included in Cohort 1. Abnormal positioning of the duodenum accompanied with clinical symptoms suggested intestinal malrotation. Surgery confirmed intestinal malrotation and revealed a chronic midgut volvulus

The overall study design was intended to assess the genetic contribution to intestinal malrotation. For this, we used two cohorts from Karolinska University Hospital, Stockholm, Sweden. Cohort 1 (*n* = 42) included adult and pediatric patients who had been treated for intestinal malrotation at Karolinska University Hospital between 2012 and 2015 and were all identified through ICD‐codes Q433 or K562 and subsequently asked to participate in the study. Diagnoses were verified through surgical records and radiological findings (Figure 1). Blood samples were collected from all participating patients, when possible also from the parents, and the patients’ medical records were reviewed. The final Cohort 1 included 20 male and 22 female patients, 40 of 42 patients had undergone abdominal surgery. Median age at inclusion was 20.5 years (range newborn‐70 years). Cohort 2 (*n* = 5) included patients with a diagnosis of intestinal malformation that had previously been investigated by clinical aCGH at the Department of Clinical Genetics at Karolinska University Hospital and were identified using search terms “intestinal malrotation” or “midgut volvulus” in the internal database of >5,000 clinically performed aCGH analyses. The database includes all clinically performed aCGH analyses, and hence also includes all healthy parental samples.

### 2.3 Array comparative genomic hybridization (aCGH) analyses

Array comparative genomic hybridization (aCGH) analyses of Cohort 1 were performed according to a previously published method (Pettersson et al., 2017) on genomic DNA derived from whole blood using standardized protocols. We used a custom high‐resolution oligonucleotide aCGH with a 2 × 400K oligonucleotide probe design targeting 1,989 genes, including all genes in the cilia proteome and known malformation syndromes. DNA labeling, hybridization, scanning, and data analysis were performed principally according to the manufacturer's recommendations as reported (Pettersson et al., 2017; Winberg et al., 2014). The aCGH data were analyzed using Cytosure Interpret Software v4.6 (OGT, Oxfordshire, UK) with following settings: three consecutive probes with deviating log2‐ratios, using log2‐ratio cutoff >0.35 for duplications and <−0.65 for deletions. Evaluation and classification of identified CNVs was performed as previously reported by us including comparison to gene dose alterations reported in public databases and the clinical database at the Department of Clinical Genetics, Karolinska University Hospital, comprising more than 3,400 patient samples analyzed by aCGH (Winberg et al., 2015). Pathogenicity classification was performed following published guidelines (Kearney, Thorland, Brown, Quintero‐Rivera, & South, 2011).

Additionally, we assessed five additional patients (Cohort 2) with a diagnosis of intestinal malrotation in the clinical aCGH database at Karolinska University Hospital between 2008 and 2018. The aCGH analyses at the Department of Clinical Genetics at Karolinska University Hospital were performed and analyzed as previously reported (Lieden, Kvarnung, Nilsson, Sahlin, & Lundberg, 2014).

## 3 RESULTS

### 3.1 Cohort clinical characteristics

Of the included 42 intestinal malrotation patients in Cohort 1, 22 (52%) had isolated intestinal malrotation. Three had secondary malrotation due to congenital diaphragmatic hernia, omphalocele, and gastroschisis, respectively. One patient had situs inversus with mirrored ventricle, left‐sided liver and gallbladder and spleen caudally on the right side. Four patients had cardiac malformations, atrial septal defect (*n* = 2), patent foramen ovale (*n* = 1), patent ductus arteriosus (*n* = 2) and heterotaxy syndrome (*n* = 1). Six patients had stenosis or atresia of the intestine: four in duodenum, one in jejunum and one had colon atresia. Two patients had urogenital malformations, one patient was diagnosed with Mayer–Rokitansky–Küster–Hauser syndrome (MIM#277000) and one patient had cryptorchidism. Additionally, one patient had cleft lip palate and five patients had CNS‐malformations, including microcephaly and holoprosencephaly. Finally, four patients had intellectual disability or developmental delay diagnosed later and three patients had cerebral palsy. At least five patients were born prematurely.

Of the five additional patients included from the clinical aCGH database (Cohort 2), none had isolated intestinal malrotation. Two patients had intellectual disability or developmental delay, one patient had autism, and one patient had delayed speech and language development. Two patients had other gastrointestinal malformations beside the intestinal malrotation: One had omphalocele and Meckel diverticulum, and one had duodenal atresia. Finally, two patients had cardiac malformations: One patient had heart valve abnormalities, and one patient had pulmonary artery stenosis.

### 3.2 Rare CNVs detected in patients with intestinal malrotation

**Figure 2:**
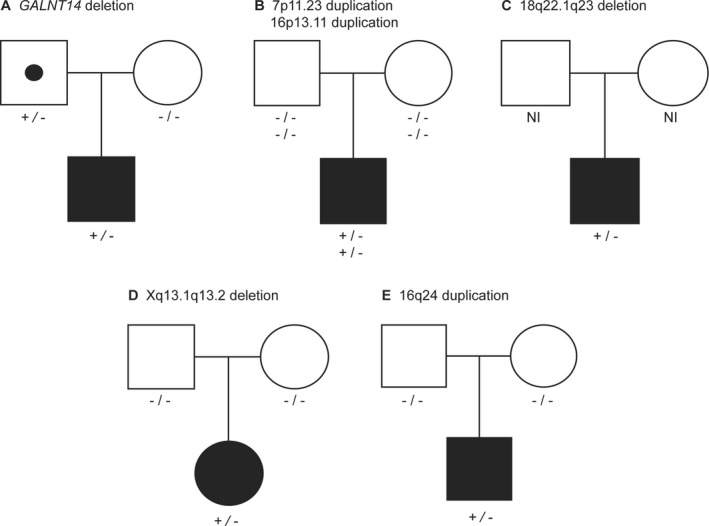
Segregation analysis of six rare CNVs found in five patients with intestinal malrotation. Parental samples were available for four patients with five rare CNVs. The intragenic deletion in *GALNT14* was inherited from the father who reported no gastrointestinal symptoms

Cohort 1 including 42 patients with intestinal malrotation was analyzed for rare CNVs using the custom aCGH, and we identified five rare CNVs in four patients (9.5%), with one patient harboring two rare CNVs (Table 1, Figure 2, Supporting information Figure S1). In addition, we queried the clinical database for cases with phenotype terms “intestinal malrotation” or “midgut volvulus” and identified five additional patients (Cohort 2), that had previously been analyzed with clinical aCGH and found an additional rare CNV (Table 1) making the total findings of rare CNV carriers 5/47 (10.6%). Clinical parameters for all five patients with rare CNVs are presented in Table 2 and detailed clinical features, and history of patients with rare CNVs are presented in Supporting information Data S1.

**Table 1:** Rare CNVs identified in five patients with intestinal malrotation

Patient	Array result	Inheritance	Hg19 CNV coordinates	Size (bp)	Affected genes/loci	Classification
1	Heterozygous deletion	Paternal	2p23.1 (31,209,654–31,332,990)^a^	123,336	*GALNT14*	Likely benign
2	Duplication	De novo	7q11.23 (72,634,873–74,142,327)	1,507,454	7q11.23 microduplication	Likely pathogenic
	Duplication	De novo	16p13.11 (15,491,492–16,292,218)	800,726	16p13.11 microduplication	VOUS
3	Heterozygous deletion	NI	18q22.1q23 (64,876,751–78,015,117)	13,138,366	18q deletion syndrome	Pathogenic
4	Heterozygous deletion	De novo	Xq13.1q13.2 (71,728,056–72,215,882)	487,826	*HDAC8* (Cornelia de Lange typ 5)	Pathogenic
5	Duplication	De novo	16q24 (86,105,735–86,105,696)^a^	1,488,327	*FOXF1*	Pathogenic

Note

NI, No information; VOUS, Variant of uncertain significance.

a Breakpoints sequenced; exact breakpoints

**Table 2:** Detailed clinical characteristics of five patients with intestinal malrotation and rare CNV findings. Patients 1–4 were included in Cohort 1, and patient 5 was included in Cohort 2

Patient	Sex	Other abdominal malformations	Neurological deficits	Additional phenotype
1	Male	No	No	–
2	Male	0.5 cm antemesenteric protuberance on colon ascendens, 1.5 cm distal to cecum	Expressional language disorder	Cryptorchidism Eczema
3	Male	Accessory spleen on descending colon Small umbilical hernia	Congenital nystagmus Delayed myelination Global developmental delay, with a dominating expressive language disturbance	Left‐sided clubfoot
4	Female	Hypertrophic pyloric stenosis Missing short gastric arteries and veins Thin gastroduodenal ligament	Delayed psychomotor development, only speaks a few words. Strabismus	Dysmorphic facial features Narrow tear ducts Growth delay Tracheomalacia Hirsutism Nevus flammeus Small hands with a proximal digit I Clinodactyly dig I Brachydactyly Hypermobility of the joints
5	Male	No	Mild intellectual disability	Short stature (−2 *SD*) Mild pulmonary artery stenosis Mildly dysmorphic facial features

Of the total of six rare CNVs detected in five patients, four CNVs were considered as likely pathogenic or pathogenic and two CNVs were classified as variants of uncertain significance (VOUS; Table 1). In patient 1, we identified an intragenic deletion of 123 kb in *GALNT14 *(MIM#608225), not previously implicated in human disease and inherited from the father. Patient 2 harbored two rare duplications, one on the 7q11.23 microduplication syndrome locus and one on 16p13.11 microduplication syndrome locus. Both duplications were de novo, and varying penetrance and phenotypes have been described in both duplications (Kirov et al., 2014), with one patient previously reported with intestinal malrotation and the 7q11.23 duplication (Morris et al., 2015). In patient 3, we identified a terminal deletion on 18q22.1 that has previously been associated with various congenital anomalies (Strathdee, Zackai, Shapiro, Kamholz, & Overhauser, 1995) and patient 4 harbored a deletion involving the *HDAC8* (MIM#300269) gene, associated with Cornelia de Lange syndrome type 5 (MIM#300882). Cornelia de Lange syndrome is known to be associated with gastrointestinal malformations, including intestinal malrotation that is seen in approximately 2.3% of cases (Kapoor, 2014). However, to our knowledge, this is the first published case of *HDAC8*‐related Cornelia de Lange syndrome with intestinal malrotation. Finally, patient 5 had a large duplication on 16q24.1 that included the *FOXF1* locus, previously implicated in syndromic forms of intestinal malrotation (Dharmadhikari et al., 2014).

All CNVs reported have been submitted to the ClinVar database (https://www.ncbi.nlm.nih.gov/clinvar/), accession numbers SCV000803395‐SCV000803400.

## 4 DISCUSSION

A few syndromes associated with intestinal malrotation have been reported with genetic etiology, as well as case reports of familial intestinal malrotation (Beaudoin et al., 2005; Martin & Shaw‐Smith, 2010; Nath & Corder, 2012; Stalker & Chitayat, 1992). To further assess the genetic contribution to intestinal malrotation, we performed a systematic screening for rare CNVs in 47 patients with intestinal malrotation and identified two variants of unknown significance and four likely pathogenic or pathogenic variants. The findings included an intragenic deletion (*GALNT14, *patient 1), three microduplications (7q11.23 and 16p13.11, patient 2; 16q24, patient 5) and two microdeletions (18q22.1‐q23, patient 3; Xq13.1‐q13.2, patient 4).

The intragenic heterozygous deletion in *GALNT14 *identified in patient 1 was inherited from a healthy father and as *GALNT14* has not previously been implicated in human disease, the deletion was considered as of uncertain significance. *GALNT14* is important during embryonal development, and its relevance in cancer and cancer therapy is being investigated (Lin et al., 2016; Mariano et al., 2015; Tsou et al., 2017). *GALNT14* overexpression increases mRNA expression of N‐cadherin (Huanna et al., 2015), which is a cell adhesion protein with an important role in asymmetric cell‐formations in the dorsal mesentery (Davis et al., 2008; Plageman, Zacharias, Gage, & Lang, 2011; Welsh et al., 2013). On the contrary, knockdown of *GALNT14* inhibits cellular migration and affects cell morphology (Wang, Yu, Zhao, Wu, & Yang, 2013). Thus, the role of *GALNT14 *in the cell morphogenesis and organization of the dorsal mesentery could be worth consideration.

In patient 2, we identified two separate microduplications, both de novo. The 7q11.23 microduplication is a known syndrome (MIM#609757) that typically leads to language and developmental delay and intellectual disability (ID) with an estimated penetrance of 44% in a developmental delay cohort (Kirov et al., 2014). Neonatal hypotonia, congenital heart defects, and cryptorchidism are common features. One reported case with the 7q11.23 microduplication had a congenital diaphragmatic hernia, one an umbilical hernia (Aa et al., 2009), and one bowel malrotation (Morris et al., 2015). The patient reported here (patient 2) has intestinal malrotation, an expressional language disorder, and cryptorchidism, all clinical features that may be caused by the 7q11.23 duplication. However, patient 2 also carries an 800 kb 16p13.11 duplication, a genomic region that is associated with neurodevelopmental disorders and congenital anomalies, with an estimated penetrance of only 8.4% in a developmental delay cohort (Kirov et al., 2014; Sahoo et al., 2011). The malformations and neurological symptoms in patient 2 may be the results of either duplication separately or by a combinatory effect. The fact that both duplications have arisen de novo supports pathogenicity for both variants; however, the available data precludes us from decisively determining the exact genetic mechanisms involved in disease.

In patient 3, a heterozygous terminal 18q22.1‐q23 deletion was identified. The patient had a complex clinical picture including congenital nystagmus, delayed myelination, global developmental delay, left‐sided clubfoot, and umbilical hernia, well concordant with the 18q deletion syndrome (MIM#601808). However, patient 3 also has an accessory spleen and intestinal malrotation which are features that have not previously been reported in patients with 18q terminal deletions (Cody et al., 1999).

In patient 4, we identified a de novo 488 kb heterozygous deletion on Xq13.1‐q13.2 involving *HDAC8*, known to be involved in X‐linked dominant Cornelia de Lange syndrome (CDLS) type 5 (MIM#300882) and classified as likely pathogenic. Patient 4 presented with intestinal malrotation, delayed psychomotor development, speech delay, strabismus, dysmorphic facial features, nevus flammeus, brachydactyly and hirsutism, which is in concordance with a Cornelia de Lange diagnosis. Intestinal malrotation is relatively rare in CDLS but is present in approximately 2.3% of cases (Kapoor, 2014). To our knowledge, this is the first reported case of CDLS type 5 presenting with intestinal malrotation, which is seen in approximately 2% of CDLS type 1 cases (Deardorff et al., 1993).

Finally, we identified a 1.5 Mb de novo duplication on 16q24 involving *FOXF1* in one patient (patient 5) presenting with intestinal malrotation, mild intellectual disability, short stature, mild pulmonary artery stenosis, and mild dysmorphic facial features. Duplications involving *FOXF1*, ranging from 15 kb to 1.7 Mb in size, have been described previously in four families with ID, speech delay, and gastrointestinal abnormalities including malrotation (Dharmadhikari et al., 2014). One patient in the previously reported *FOXF1* duplications paper harbored a duplication similar in size and location to our patient, and also presented with similar neurological features including borderline intellectual disability, but no intestinal malrotation or cardiac defects (Dharmadhikari et al., 2014).

Out of the total 42 patients in Cohort 1, 22 had isolated intestinal malrotation. Only one patient with isolated intestinal malrotation harbored a rare CNV (*GALNT14, *patient 1), which was inherited from a healthy parent. All four patients with likely pathogenic or pathogenic findings had intestinal malrotation and additional malformations or deficits, suggesting that genetic testing in patients with intestinal malrotation should be recommended in such cases. In two of the known syndromes that we identified in our cohort (18q deletion syndrome, patient 3 and Cornelia de Lange type 5, patient 4), intestinal malrotation has not previously been associated with the syndrome but could be an underreported rare phenotype.

The median age at inclusion in this study is high compared to the typical patient with intestinal malrotation. The older patients typically had symptoms since early childhood but were diagnosed later in life. The response rates were higher among the adult patients at the time of the study and thus lead to a skewed median age. This weakens the study, though additional malformations and comorbidities have been discovered since all are not always evident at birth. All patients had symptomatic intestinal malrotation.

The small cohorts are a limitation of the present study and also the large age span as mentioned previously might be a limitation if you regard that as different phenotype severity. Thus further genetic studies on larger cohorts are necessary to fully understand the impact of rare CNVs in intestinal malrotation, as aCGH is a powerful tool to identify disease‐causing genes in both isolated and syndromic gastrointestinal malformations (Dworschak et al., 2015; Genesio et al., 2018; Tsai et al., 2015).

In conclusion, we identified likely pathogenic or pathogenic CNVs in 4/47 (8.5%) patients with intestinal malrotation. All cases with pathogenic CNVs had intestinal malrotation‐related malformations and/or neurological symptoms. These findings suggest that genetic investigation with CNV screening is indicated in cases with intestinal malrotation and additional extra‐gastrointestinal phenotypes. Finally, we present here, to our knowledge, the first cases of CDLS type 5 and 18q terminal deletion syndrome with intestinal malrotation.

## CONFLICTS OF INTEREST

The authors declare no conflicts of interest.
