# Supplementary figures and images for: Rare copy number variants contribute pathogenic alleles in patients with intestinal malrotation
Source: Mol Genet Genomic Med. 2019 Jan 10;7(3):e549. doi: 10.1002/mgg3.549 (PMC6418355; doi:10.1002/mgg3.549)

Supplementary Figure 1

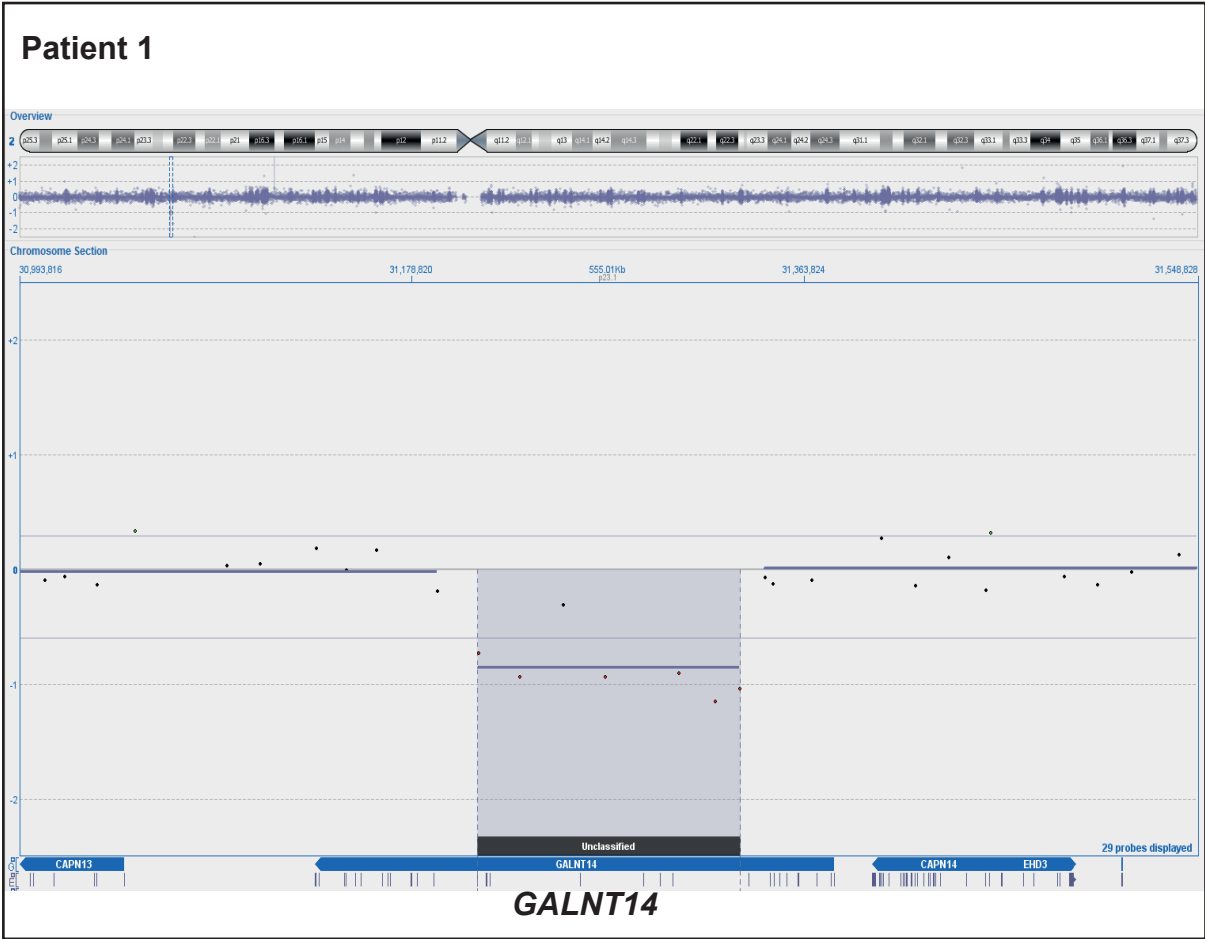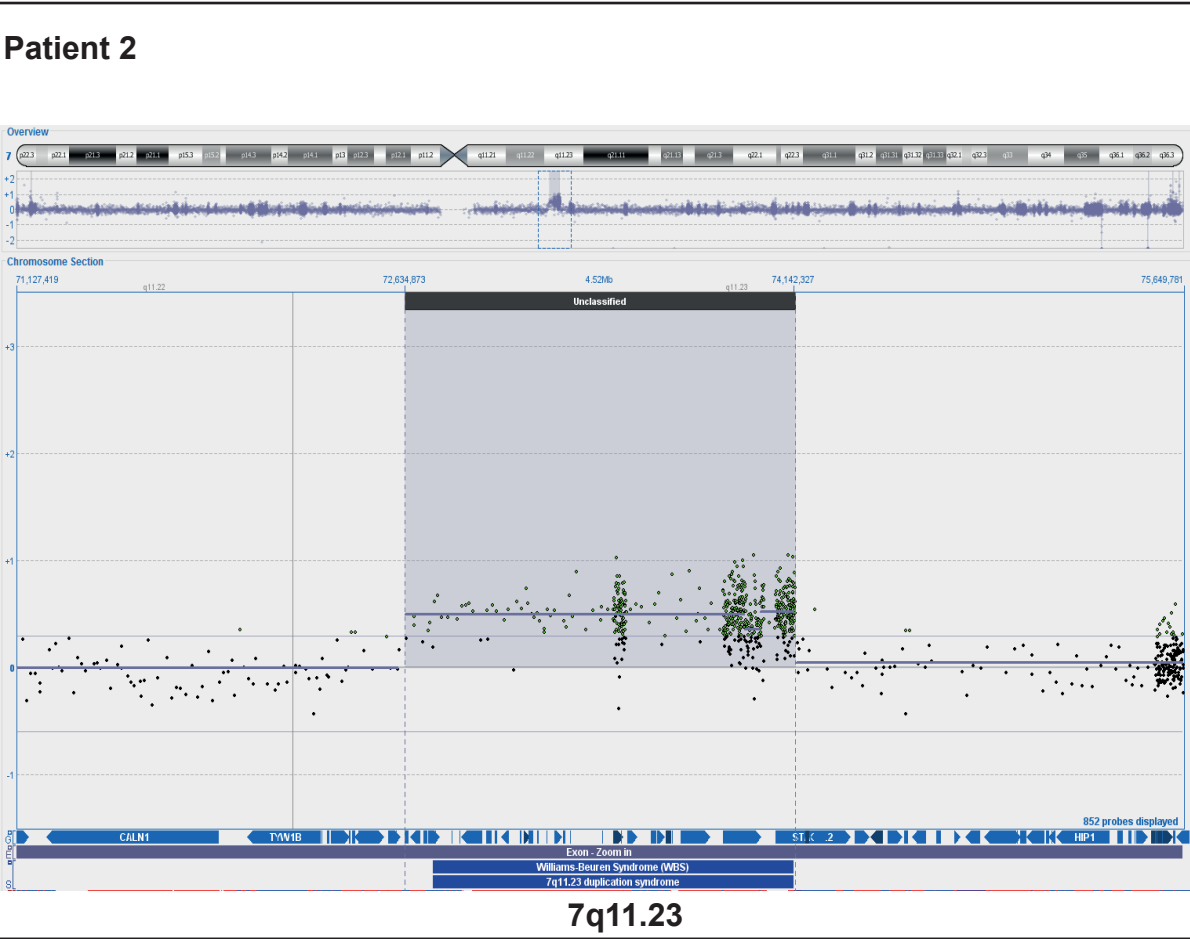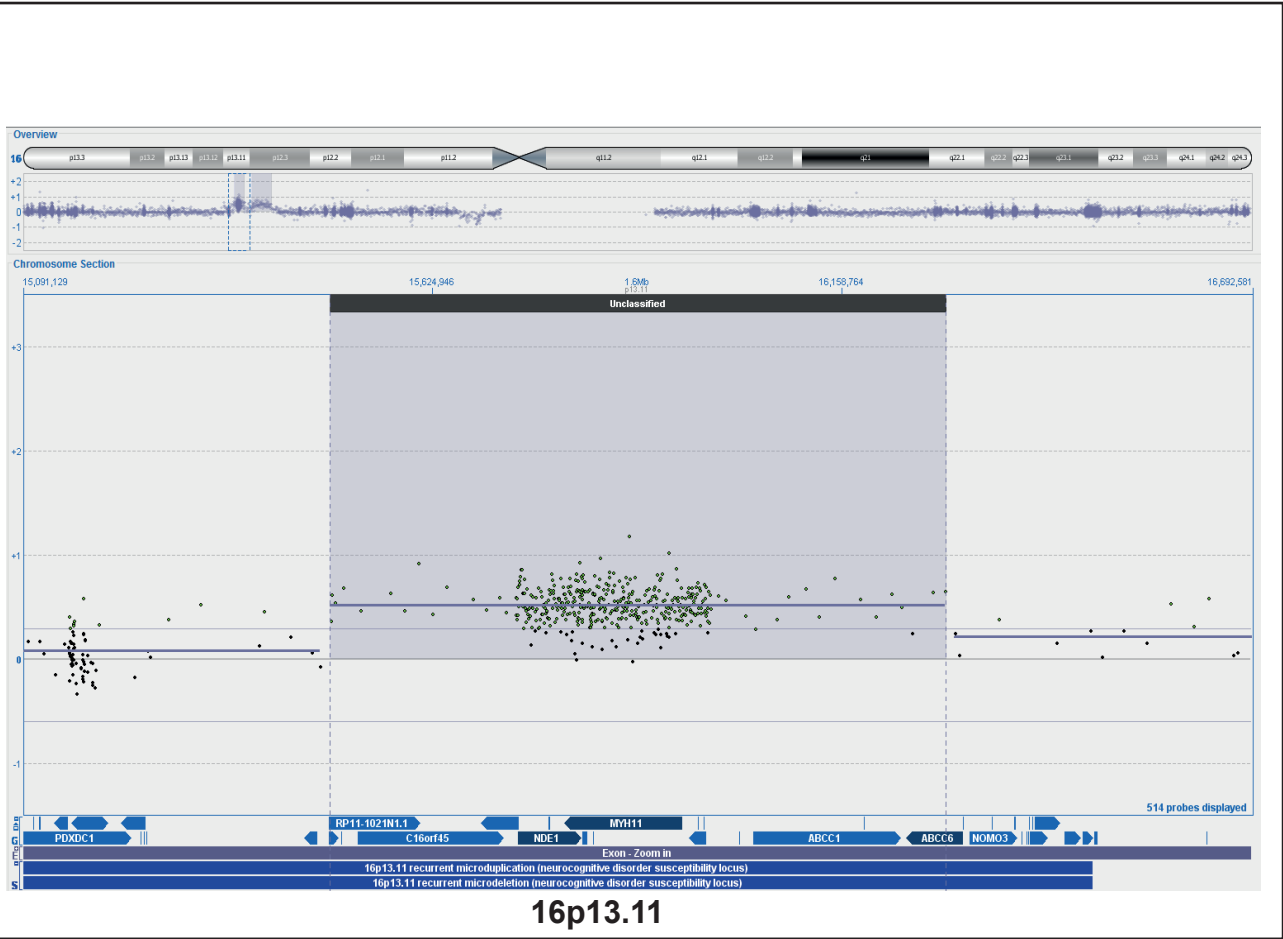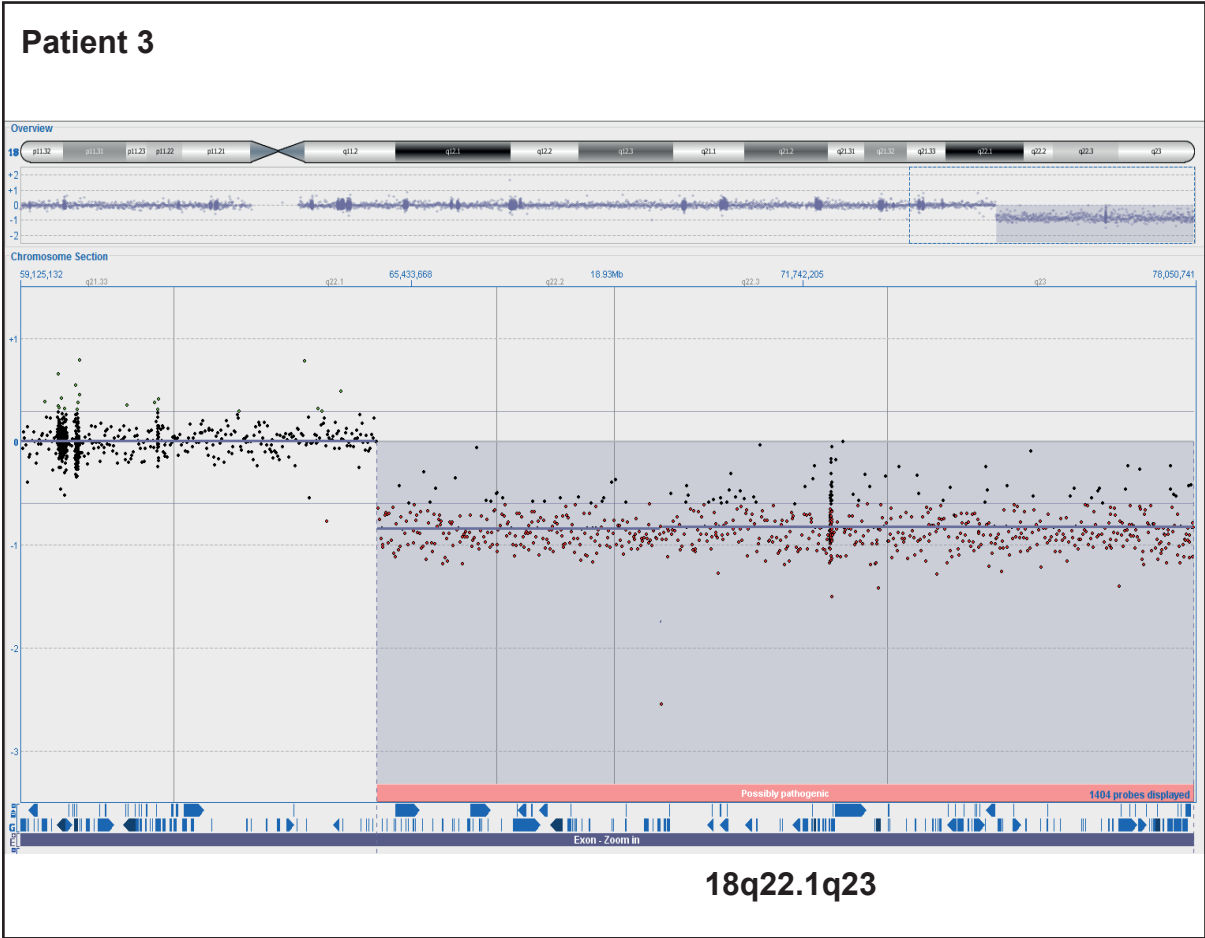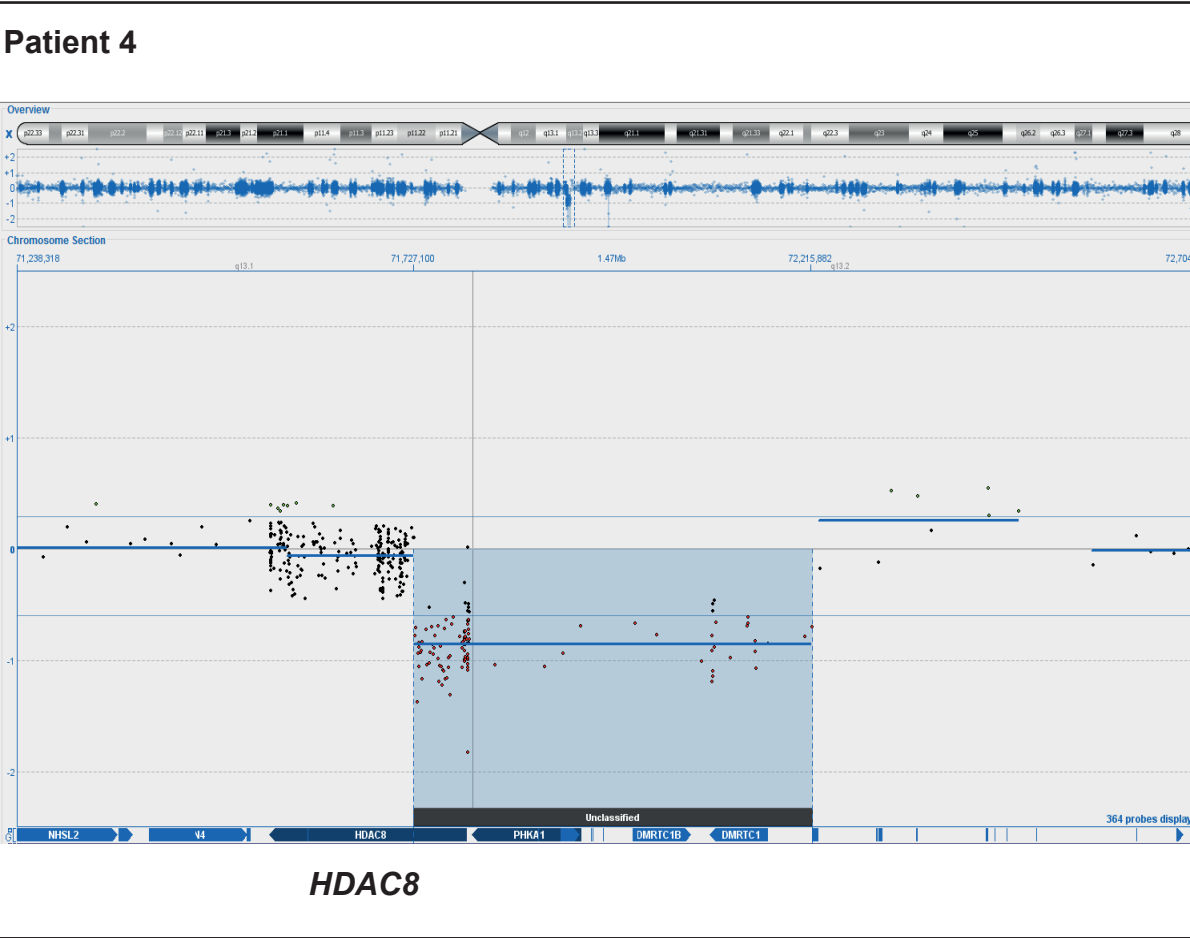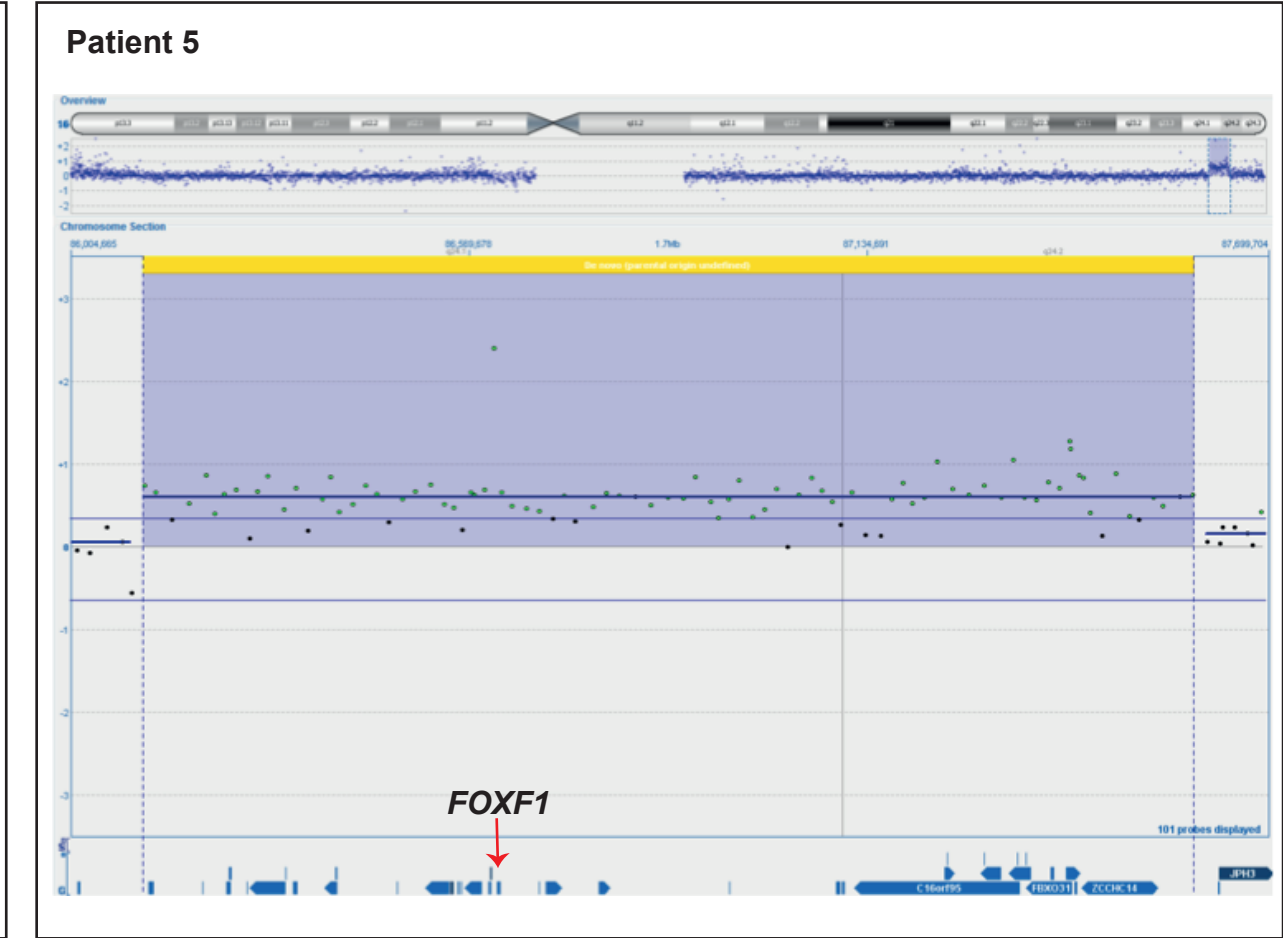

Supplement: Supplementary file 1 [file MGG3-7-na-s001.pdf]
