# Supplementary material for: Rare copy number variants contribute pathogenic alleles in patients with intestinal malrotation
Source: Mol Genet Genomic Med. 2019 Jan 10;7(3):e549. doi: 10.1002/mgg3.549 (PMC6418355; doi:10.1002/mgg3.549)
Supplement: Supplementary file 2 [file MGG3-7-na-s002.docx]

**Supplementary File 1**

**Detailed clinical characteristics of patients with rare CNVs**

**Patient 1** had an isolated intestinal malrotation. Due to his intestinal malrotation, the patient had difficulties eating full meals already during childhood and later he often suffered from abdominal pain. He was operated as an emergency at age 35 years due to caecal volvulus that was twisted two leaps around its axis. An ileocaecal resection was performed and diagnosis of intestinal malrotation was made but not surgically treated. Computed Tomography with double contrast, confirmed a pathologic picture of intestinal malrotation with the duodeno-jejunal junction caudally on the right side of the midline, not passing the spine to the left, all small intestine located on the right side and most of colon situated on the left. Superior mesenteric artery is located to the right of superior mesenteric vein. Segregation analysis showed that the deletion was inherited from the healthy father.

**Patient 2** was operated due to intestinal malrotation 10 days after birth, diagnosis was preoperatively established with an upper gastrointestinal series and confirmed with surgery. He has had surgery for right-sided cryptorchidism and at five years of age he is being investigated for expressional language disorder.

**Patient 3** had bile vomiting since birth and failure to thrive at two months after birth. He was diagnosed with intestinal malrotation with an upper gastrointestinal study when he was 15 months old and operated with a good result that also confirmed the diagnosis. Magnetic resonance imaging of the brain performed due to congenital nystagmus and intermittent strabismus showed delayed myelination at the age of one year. At five years of age he exhibited a global developmental delay with expressive language disturbance. Other malformations were a small accessory spleen and left sided clubfoot.

**Patient 4** had growth retardation at gestational week 29 and was born full time with tracheomalacia and a birth weight of 2300g (-2 SD). When she was five weeks old, she was operated due to gastric volvulus and malrotation of the intestines. In addition, there was a hypertrophic pyloric stenosis and missing short gastric arteries and veins. At six years of age, she demonstrated psychomotor and growth delay including mild hearing loss, severe visual impairment on one eye and severe speaking difficulties. She has almond shaped eyes, like her father, micrognathia and a slightly abnormal shape of the skull and hypermobility in the joints. Her chest cage is flat and she has pointy heals and hairy legs and back. Other dysmorphic features were narrow tear ducts, hooded eyelids, flat nasal bridge, smooth philtrum, sparse areas between the teeth, hirsuitism, naevus flammeus, small hands with a proximal thumb, clinodactyly dig 5 and brachydactyly. She also has hypermobility in the joints. Ultrasound of the brain is normal and she has never had any epileptic seizures.

**Patient 5** was a 13 year-old boy with major difficulties in school, previous aortic stenosis, and previous emergency surgery for intestinal malrotation at age 12. Additional phenotypes include failure to thrive and mildly dysmorphic facial features.
